# Intramuscular adipose tissue in the quadriceps is more strongly related to recovery of activities of daily living than muscle mass in older inpatients

**DOI:** 10.1002/jcsm.12713

**Published:** 2021-05-16

**Authors:** Naoki Akazawa, Masaki Kishi, Toshikazu Hino, Ryota Tsuji, Kimiyuki Tamura, Akemi Hioka, Hideki Moriyama

**Affiliations:** ^1^ Department of Physical Therapy, Faculty of Health and Welfare Tokushima Bunri University Yamashiro‐cho Tokushima Tokushima Japan; ^2^ Department of Rehabilitation Kasei Tamura Hospital Wakayama Wakayama Japan; ^3^ Life and Medical Sciences Area, Health Sciences Discipline Kobe University Kobe Hyogo Japan

**Keywords:** Intramuscular adipose tissue, Activities of daily living, Older inpatients, Muscle mass, Quadriceps

## Abstract

**Background:**

The relationship between intramuscular adipose tissue at admission and recovery of activities of daily living (ADL) remains unclear. This study aimed to examine the relationship between intramuscular adipose tissue in the quadriceps at admission and recovery of ADL in older inpatients.

**Methods:**

This prospective study included 404 inpatients aged ≥65 years (54.7% female). Recovery of ADL during hospital stay was assessed using the Barthel Index (BI) score at discharge, BI score change, and BI efficiency. Higher BI at discharge, BI score change, and BI efficiency indicate more improvement in ADL. Intramuscular adipose tissue and muscle mass of the quadriceps were assessed using echo intensity and muscle thickness on ultrasound images, respectively. Multiple regression analysis was performed to identify factors independently associated with BI score at discharge, BI score change, and BI efficiency. The independent variables were BI score at admission, echo intensity and muscle thickness of the quadriceps, age, sex, number of medications, C‐reactive protein concentration, updated Charlson Comorbidity Index score, Food Intake Level Scale, Geriatric Nutritional Risk Index score, days from onset disease, length of hospital stay, number of units of rehabilitation therapy, and subcutaneous fat thickness of the thigh.

**Results:**

The medians (inter‐quartile range) of the BI score at discharge, BI score change, and BI efficiency were 60.0 (35.0–80.0), 10.0 (0.0–25.0), and 0.11 (0.00–0.37), respectively. The median (inter‐quartile range) of the length of hospital stay (days) and days from onset disease were 58.0 (39.0–92.0) and 79.0 (49.0–112.0), respectively. Quadriceps echo intensity was independently and significantly associated with the BI score at discharge (*β* = −0.13, *P* < 0.01), BI score change (*β* = −0.23, *P* < 0.01), and BI efficiency (*β* = −0.21, *P* < 0.01). Quadriceps thickness was not independently and significantly associated with the BI score at discharge (*β* = −0.02, *P* = 0.68), BI score change (*β* = −0.02, *P* = 0.79), and BI efficiency (*β* = 0.03, *P* = 0.67).

**Conclusions:**

Our study indicates that greater intramuscular adipose tissue in the quadriceps at admission is more strongly related to worse recovery of ADL than less muscle mass in older inpatients. Greater intramuscular adipose tissue in the quadriceps in older inpatients is considered to be a predictor of worse recovery of ADL, and intervening for greater intramuscular adipose tissue may be important for improving ADL in older inpatients.

## Introduction

Ageing is known to be associated with an increase in intramuscular adipose tissue.^1^, ^2^, ^3^, ^4^ Furthermore, greater intramuscular adipose tissue is more strongly associated with decreased muscle strength^5^, ^6^, ^7^; decreased sit‐up–sit‐down,^5^, ^8^ stair ascent–stair descent,^9^ and gait abilities^6^, ^10^, ^11^; onset hip fracture^12^; and mortality^13^, ^14^ than less muscle mass and is considered a serious problem in old age. In fact, the European Working Group of Sarcopenia in Older People 2 suggested the importance of assessing not only muscle mass but also intramuscular adipose tissue when evaluating muscle quality.^15^

The levels of perilipin proteins, such as perilipin2, an indicator of intramuscular adipose tissue, have been reported to increase with age and inactivity.^16^ In addition, a higher level of perilipin2 expression is also associated with reduced muscle mass and muscle strength.^16^ In support of these findings, it has been reported that the amount of intramuscular adipose tissue in the quadriceps in older frail individuals and older inpatients is greater than that of healthy persons matched for age, sex, and body mass index (BMI).^11^, ^17^ In addition, greater intramuscular adipose tissue in the quadriceps in older inpatients is related to decreased gait independence^11^ and swallowing function^18^ than less muscle mass.

A more recent cross‐sectional study^19^ reported that declines in activities of daily living (ADL) were more strongly associated with greater intramuscular adipose tissue in the quadriceps in older inpatients than with less muscle mass. However, the relationship between intramuscular adipose tissue at admission and recovery of ADL remains unclear. Understanding this relationship is essential for the development of an effective approach to improve ADL. This study aimed to examine the relationship between intramuscular adipose tissue in the quadriceps at admission and recovery of ADL in older inpatients.

## Materials and methods

### Study design and participants

This prospective study included older inpatients who were referred to the Department of Rehabilitation at Kasei Tamura Hospital between March 2017 and June 2020. This hospital has subacute and convalescent rehabilitation wards. Patients aged <65 years or those who lacked data were excluded from the study. A total of 455 inpatients were recruited using convenience sampling. Of these, 51 patients aged <65 years (*n* = 33) or who lacked necessary data (*n* = 18) were excluded. Consequently, 404 inpatients participated in this study. Rehabilitation therapy, including physical therapy, occupational therapy, and speech and swallowing therapy, was administered to all participants during hospitalization. All participants or their guardians provided informed consent prior to the study, and the study was approved by the ethics committee of our institution.

### Outcome measures

The primary outcome was recovery of ADL during the hospital stay. We also measured the characteristics of the participants at admission including, disease, intramuscular adipose tissue and muscle mass of the quadriceps, age, sex, body weight, height, BMI, subcutaneous fat mass of the thigh, ADL, swallowing function, nutritional status, inflammation, co‐morbidities, number of medications, and number of units of rehabilitation therapy (one unit of rehabilitation therapy = 20 min). The length of hospital stay (days) and days from onset disease were measured at discharge. The length of hospital stay (days) was assessed based on the hospitalization period at Kasei Tamura Hospital. The majority of older inpatients at our hospital were admitted from another acute‐phase hospital. In these patients, the days from onset disease were assessed as the total length of stay (days) in both hospitals.

### Measurement of recovery of activities of daily living

ADL​ were assessed using the Barthel Index (BI).^20^ Recovery of ADL during hospital stay was assessed based on the BI score at discharge, BI score change, and BI efficiency. The BI is widely used in clinical settings and includes ordinal assessment (0–100 points).^20^ Lower BI scores indicate poor ability to perform ADL. The BI consists of 10 items: (i) feeding, (ii) moving from a wheelchair to a bed and from a bed to a wheelchair, (iii) grooming, (iv) toilet action, (v) bathing, (vi) walking on a level surface, (vii) going up and down stairs, (viii) dressing, (ix) bowel continence, and (x) bladder continence.^20^ The BI score change and BI efficiency were calculated using the following equations: BI score change = BI score at discharge − BI score at admission and BI efficiency = BI score change∕length of hospital stay (days). A higher BI score change and BI efficiency indicated a greater improvement in the ability to perform ADL. The BI score at discharge of the deceased participants was 0.

### Measurements of intramuscular adipose tissue and muscle mass of the quadriceps and subcutaneous fat mass of the thigh

Transverse ultrasound images were obtained using a B‐mode ultrasound system (NanoMaxx; SonoSite Japan, Tokyo, Japan) with a linear‐array probe (L25n/13–6 MHz; Nanomaxx; SonoSite Japan). The intramuscular adipose tissue and muscle mass of the rectus femoris and vastus intermedius of all participants were assessed based on the echo intensity and muscle thickness.^1^, ^2^, ^3^, ^4^, ^5^, ^6^, ^7^, ^8^, ^10^, ^11^, ^18^, ^19^, ^21^, ^22^, ^23^, ^24^ The validity of intramuscular adipose tissue and muscle mass measurements using ultrasound has been confirmed in recent studies using magnetic resonance imaging.^25^, ^26^, ^27^ Images of the rectus femoris and vastus intermedius were obtained at 30% of the distance from the anterior superior iliac spine to the proximal end of the patella.^4^, ^6^, ^11^, ^18^, ^19^, ^24^ The participants lay in the supine position with their lower limbs relaxed, while a water‐soluble transmission gel was applied to the skin surface of the thigh. The probe was pressed perpendicularly and lightly against the skin to prevent muscle deformation. All ultrasound images were recorded by the same investigator, who had sufficient training in echo intensity and muscle thickness measurements. Echo intensity was measured in one region of interest in the rectus femoris and vastus intermedius; the region selected included the maximum possible regions, while avoiding the bone and surrounding fascia.^4^, ^6^, ^11^, ^18^, ^19^, ^24^ To standardize all echo intensity measurements, the gain status was normalized to the initial setting of the ultrasound system. In addition, the image depth was uniformed at 60 mm for all echo intensity and muscle thickness measurements. The rectus femoris thickness was determined as the distance between the superficial adipose tissue–muscle interface and the deep muscle–muscle interface,^4^, ^6^, ^11^, ^18^, ^19^, ^24^ and that of the vastus intermedius was determined as the distance between the superficial muscle–muscle interface and the bone–muscle interface.^4^, ^6^, ^11^, ^18^, ^19^, ^24^ Echo intensity and muscle thickness were measured using ImageJ 1.49 software (National Institutes of Health, Bethesda, MD, USA).^1^, ^2^, ^3^, ^4^, ^5^, ^6^, ^7^, ^8^, ^11^, ^18^, ^19^, ^21^, ^22^, ^24^, ^26^, ^27^ Echo intensity was determined by performing a computer‐assisted 8 bit greyscale analysis, and the mean echo intensity of the regions of interest was expressed as a value from 0 (black) to 255 (white).^1^, ^2^, ^3^, ^4^, ^5^, ^6^, ^7^, ^8^, ^11^, ^18^, ^19^, ^21^, ^22^, ^23^, ^24^, ^26^, ^27^ Higher echo intensity indicates greater intramuscular adipose tissue.^23^

The echo intensity of the quadriceps was calculated as the mean echo intensity of the rectus femoris and vastus intermedius. The mean echo intensity of the right and left quadriceps was used in the analysis. The sum of the thickness of the rectus femoris and vastus intermedius was used as a measure of quadriceps thickness. The mean thickness of the right and left quadriceps was used in the analysis. The methods used for measuring the echo intensity and muscle thickness of the rectus femoris and vastus intermedius in our study group have been reported to have high reliability [intraclass correlation coefficients (1.1) = 0.857–0.959].^24^ The subcutaneous fat mass of the thigh was assessed based on the subcutaneous fat thickness. Subcutaneous fat thickness was determined as the distance between the dermis and adipose tissue interface and the muscle–adipose tissue interface.^4^, ^6^, ^11^, ^18^, ^19^, ^24^ The mean subcutaneous fat thickness of the right and left thighs was used in the analysis.

### Measures of other characteristics

Swallowing function was assessed using the Food Intake Level Scale (FILS).^28^ The FILS is a 10‐point observer‐rated scale, with higher values indicating better swallowing function. The inflammatory status was assessed by analysing C‐reactive protein concentration. Nutritional status was assessed using the Geriatric Nutritional Risk Index (GNRI) score.^29^ The GNRI score was calculated using the following formula: GNRI score = (1.489 × serum albumin [g/dL]) + (41.7 × body weight [kg]∕ideal body weight).^29^ The ideal body weight was defined based on a BMI of 22.0 kg/m^2^.^30^ If the body weight∕ideal body weight was ≥1.0, the value was recorded as 1.^29^ Co‐morbidities were evaluated using the updated Charlson Comorbidity Index (UCCI) score.^31^

### Statistical analysis

All statistical analyses were conducted using SPSS Version 24 software (IBM SPSS Japan, Tokyo, Japan). Variables were assessed for normality using the Shapiro–Wilk test. Parametric data are reported as mean ± standard deviation, whereas non‐parametric data are expressed as median [inter‐quartile range (IQR)]. Relationships between BI score at discharge, BI score change, BI efficiency, and quadriceps echo intensity and thickness were assessed using Kendall's *τ* rank correlation coefficient. Multiple regression analysis was performed to identify the factors independently associated with BI score at discharge, BI score change, and BI efficiency. The independent variables were the BI score at admission, echo intensity and muscle thickness of the quadriceps, age, sex, number of medications, C‐reactive protein concentration, UCCI score, FILS, GNRI score, days from onset disease, length of hospital stay, number of units of rehabilitation therapy, and subcutaneous fat thickness of the thigh. When BI efficiency was a dependent variable, the length of hospital stay was excluded from the regression model. The echo intensity is reportedly influenced by subcutaneous fat thickness.^32^ Based on this finding, we included subcutaneous fat thickness of the thigh as an independent variable. In all multiple regression analyses, male and female inpatients were coded as 1 and 0, respectively. To assess multicollinearity, we used the variance inflation factor. A variance inflation factor value of more than 10 was considered as the presence of multicollinearity. In addition, to confirm whether sex differences were observed in the relationships between quadriceps echo intensity and BI score at discharge, BI score change, and BI efficiency, the same multiple regression analyses were separately performed in male (*n* = 183) and female (*n* = 221) models. A *P*‐value of <0.05 was considered to indicate statistical significance. In addition, we calculated the effect size (*f* ^2^) of the multiple regression analysis using the following equation: *R* ^2^∕(1 − *R* ^2^).^33^ The statistical power of the analysis based on *f* ^2^, an alpha error of 0.05, total sample size, and number of predictor variables was calculated using G*Power Version 3.1.9.2 (Heinrich‐Heine‐Universität Düsseldorf, Düsseldorf, Germany).

### Sample size calculation

A recent cross‐sectional study^19^ reported that the effect size (*f* ^2^) of the multiple regression analysis for the motor Functional Independence Measure score at admission was 0.580 (in this multiple regression analysis, the quadriceps echo intensity and the other five variables were independently and significantly associated with the motor Functional Independence Measure score). We expected to observe similar effect sizes in the multiple regression analysis for the BI score at discharge, BI score change, and BI efficiency in this study. A priori sample size calculation with an effect size (*f* ^2^) of 0.580, power of 0.99, alpha error of 0.05, and number of predictors of 14 indicated that a sample size of at least 75 participants was required. Sample size calculations were conducted using G*Power Version 3.1.9.2.

## Results

### Characteristics of the participants

Diseases found among the participants were stroke (*n* = 60), hip fracture (*n* = 49), compression fracture (*n* = 46), pubic fracture (*n* = 7), other fracture (*n* = 24), pneumonia (*n* = 64), heart disease (*n* = 25), spinal cord disease (*n* = 15), urinary tract infection (*n* = 12), and others (*n* = 102). The medians (IQR) of the BI score at discharge, BI score change, and BI efficiency were 60.0 (35.0–80.0), 10.0 (0.0–25.0), and 0.11 (0.00–0.37), respectively. The medians (IQR) of the length of hospital stay (days) and days from onset disease were 58.0 (39.0–92.0) and 79.0 (49.0–112.0), respectively. Fifteen participants died during their hospital stay. *Table* 1 shows the characteristics of the participants on admission.

**Table 1:** Participants' characteristics at admission (*n* = 404)

Characteristics	Median (25th–75th percentile), *n* (%), or mean ± standard deviation
Age (years)	83.0 (77.0–88.0)
Sex (male/female)	183 (45.3)/221 (54.7)
Body weight (kg)	46.4 (39.5–53.9)
Height (cm)	152.0 (146.3–161.0)
Body mass index (kg/m^2^)	19.9 (17.5–22.5)
Quadriceps echo intensity (0–255)^a^	84.7 ± 21.7
Quadriceps thickness (cm)	1.2 (0.9–1.5)
Subcutaneous fat thickness of the thigh (cm)	0.4 (0.3–0.5)
Barthel Index score at admission	40.0 (20.0–60.0)
Food Intake Level Scale	8.0 (7.0–9.0)
Geriatric Nutritional Risk Index score	87.6 (80.0–93.8)
C‐reactive protein (mg/dL)	0.5 (0.4–1.7)
Serum albumin (g/dL)	3.4 (3.0–3.7)
Updated Charlson Comorbidity Index score	2.0 (0.0–3.0)
Number of medications	7.0 (5.0–10.0)
Number of rehabilitation therapy (units/day)	3.0 (2.0–4.0)

^a^ Greyscale.

### Quadriceps echo intensity and thickness were significantly associated with Barthel Index score at discharge, Barthel Index score change, and Barthel Index efficiency

*Table* 2 shows the correlation coefficients between the BI score at discharge, BI score change, BI efficiency, and quadriceps echo intensity and thickness. Quadriceps echo intensity was negatively and significantly associated with BI score at discharge (*τ* = −0.24, *P* ≤ 0.01), BI score change (*τ* = −0.16, *P* ≤ 0.01), and BI efficiency (*τ* = −0.17, *P* ≤ 0.01). Quadriceps thickness was positively and significantly associated with BI score at discharge (*τ* = 0.22, *P* ≤ 0.01), BI score change (*τ* = 0.11, *P* ≤ 0.01), and BI efficiency (*τ* = 0.13, *P* ≤ 0.01).

**Table 2:** Relationships between Barthel Index score at discharge, Barthel Index score change, Barthel Index efficiency, and quadriceps echo intensity and thickness (*n* = 404)

Variables	Barthel Index score at discharge	Barthel Index score change	Barthel Index efficiency
Quadriceps echo intensity	−0.24**	−0.16**	−0.17**
Quadriceps thickness	0.22**	0.11**	0.13**

** *P* < 0.01.

### Quadriceps echo intensity was independently and significantly associated with Barthel Index score at discharge than muscle thickness

The results of the multiple regression analysis for BI score at discharge are shown in *Table* 3. No multicollinearity was observed between the independent variables in the multiple regression analysis. Quadriceps echo intensity, BI score at admission, sex, UCCI score, FILS, GNRI score, and length of hospital stay were independently and significantly associated with BI score at discharge (*R* ^2^ = 0.672, *f* ^2^ = 2.049, statistical power = 1.000). Quadriceps thickness was not independently and significantly associated with BI score at discharge.

**Table 3:** Relationships between Barthel Index score at discharge and other variables (*n* = 404)

Variables	*B*	SE	95% confidence interval of *B*	*β*	VIF	*P*‐value
Quadriceps echo intensity	−0.18	0.06	−0.30, −0.07	−0.13	2.25	<0.01
Quadriceps thickness	−1.21	2.89	−6.88, 4.47	−0.02	2.57	0.68
Subcutaneous fat thickness of the thigh	−2.24	4.40	−10.89, 6.41	−0.02	1.48	0.61
Barthel Index score at admission	0.73	0.05	0.64, 0.82	0.61	1.69	<0.01
Age	0.03	0.13	−0.22, 0.29	0.01	1.27	0.81
Sex	−4.50	1.86	−8.16, −0.84	−0.08	1.19	0.02
Number of medications	−0.36	0.24	−0.82, 0.11	−0.05	1.12	0.13
C‐reactive protein	−0.36	0.32	−1.00, 0.27	−0.04	1.16	0.26
Updated Charlson Comorbidity Index score	−1.85	0.41	−2.65, −1.05	−0.14	1.11	<0.01
Food Intake Level Scale	2.59	0.58	1.46, 3.73	0.16	1.53	<0.01
Geriatric Nutritional Risk Index score	0.24	0.11	0.02, 0.46	0.09	1.83	0.03
Days from onset disease	−0.04	0.03	−0.10, 0.01	−0.09	4.35	0.13
Length of hospital stay	0.11	0.03	0.05, 0.18	0.21	4.17	<0.01
Number of rehabilitation therapy	1.12	0.57	−0.01, 2.24	0.06	1.20	0.05

*B*, partial regression coefficient; SE, standard error; VIF, variance inflation factor; *β*, standardized partial regression coefficient.

### Quadriceps echo intensity was independently and significantly associated with Barthel Index score change than muscle thickness

The results of the multiple regression analysis for BI score change are shown in *Table* 4. No multicollinearity was observed between the independent variables in the multiple regression analysis. Quadriceps echo intensity, BI score at admission, sex, UCCI score, FILS, GNRI score, length of hospital stay, and number of units of rehabilitation therapy were independently and significantly associated with BI score change (*R* ^2^ = 0.228, *f* ^2^ = 0.295, statistical power = 1.000). The quadriceps thickness was not independently and significantly associated with BI score change.

**Table 4:** Relationships between Barthel Index score change and other variables (*n* = 404)

Variables	*B*	SE	95% confidence interval of *B*	*β*	VIF	*P*‐value
Quadriceps echo intensity	−0.22	0.06	−0.34, −0.09	−0.23	2.25	<0.01
Quadriceps thickness	−0.84	3.08	−6.90, 5.22	−0.02	2.57	0.79
Subcutaneous fat thickness of the thigh	−3.06	4.70	−12.29, 6.18	−0.04	1.48	0.52
Barthel Index score at admission	−0.27	0.05	−0.37, −0.18	−0.32	1.69	<0.01
Age	0.08	0.14	−0.19, 0.35	0.03	1.27	0.57
Sex	−5.25	1.99	−9.16, −1.34	−0.13	1.19	0.01
Number of medications	−0.44	0.25	−0.93, 0.06	−0.08	1.12	0.08
C‐reactive protein	−0.25	0.35	−0.92, 0.43	−0.03	1.16	0.48
Updated Charlson Comorbidity Index score	−1.79	0.43	−2.64, −0.94	−0.20	1.11	<0.01
Food Intake Level Scale	2.59	0.62	1.38, 3.81	0.23	1.53	<0.01
Geriatric Nutritional Risk Index score	0.25	0.12	0.02, 0.48	0.13	1.83	0.04
Days from onset disease	−0.05	0.03	−0.11, 0.02	−0.14	4.35	0.14
Length of hospital stay	0.11	0.03	0.04, 0.17	0.29	4.17	<0.01
Number of rehabilitation therapy	1.46	0.61	0.26, 2.67	0.12	1.20	0.02

*B*, partial regression coefficient; SE, standard error; VIF, variance inflation factor; *β*, standardized partial regression coefficient.

### Quadriceps echo intensity was independently and significantly associated with Barthel Index efficiency than muscle thickness

The results of the multiple regression analysis for BI efficiency are shown in *Table* 5. No multicollinearity was observed between the independent variables in the multiple regression analysis. The quadriceps echo intensity, BI score at admission, sex, number of medications, UCCI score, FILS, and length of hospital stay were independently and significantly associated with BI efficiency (*R* ^2^ = 0.141, *f* ^2^ = 0.164, statistical power = 0.999). Quadriceps thickness was not independently and significantly associated with BI efficiency.

**Table 5:** Relationships between Barthel Index efficiency and other variables (*n* = 404)

Variables	*B*	SE	95% confidence interval of *B*	*β*	VIF	*P*‐value
Quadriceps echo intensity	−0.00	0.00	−0.01, −0.00	−0.21	2.23	<0.01
Quadriceps thickness	0.03	0.06	−0.09, 0.14	0.03	2.57	0.67
Subcutaneous fat thickness of the thigh	−0.04	0.09	−0.22, 0.13	−0.03	1.47	0.64
Barthel Index score at admission	−0.00	0.00	−0.00, −0.00	−0.16	1.65	<0.01
Age	0.00	0.00	−0.01, 0.01	0.01	1.27	0.86
Sex	−0.08	0.04	−0.15, −0.00	−0.10	1.19	0.04
Number of medications	−0.01	0.01	−0.02, −0.00	−0.11	1.12	0.04
C‐reactive protein	−0.01	0.01	−0.02, 0.01	−0.05	1.16	0.29
Updated Charlson Comorbidity Index score	−0.03	0.01	−0.04, −0.01	−0.16	1.12	<0.01
Food Intake Level Scale	0.03	0.01	0.01, 0.05	0.14	1.53	0.02
Geriatric Nutritional Risk Index score	0.00	0.00	−0.00, 0.01	0.03	1.83	0.69
Days from onset disease	−0.00	0.00	−0.00, 0.00	−0.11	1.14	0.03
Number of rehabilitation therapy	0.01	0.01	−0.01, 0.04	0.06	1.18	0.22

*B*, partial regression coefficient; SE, standard error; VIF, variance inflation factor; *β*, standardized partial regression coefficient.

### Quadriceps echo intensity was independently and significantly associated with Barthel Index score change and Barthel Index efficiency but not Barthel Index score at discharge in the male model

The results of multiple regression analysis in the male model for BI score at discharge, BI score change, and BI efficiency are shown in Supporting Information, *Tables* *S1* *–* *S3*. No multicollinearity was observed between the independent variables in all multiple regression analyses. Quadriceps echo intensity was independently and significantly associated with BI score change and BI efficiency. There was no relationship between quadriceps echo intensity and BI score at discharge. Quadriceps thickness was not independently and significantly associated with BI score at discharge, BI score change, or BI efficiency.

### Quadriceps echo intensity was independently and significantly associated with Barthel Index score at discharge, Barthel Index score change, and Barthel Index efficiency in the female model

The results of multiple regression analysis in the female model for BI score at discharge, BI score change, and BI efficiency are shown in *Tables* *S4*–*S6*. No multicollinearity was observed between the independent variables in all multiple regression analyses. Quadriceps echo intensity was independently and significantly associated with BI score at discharge, BI score change, and BI efficiency. The quadriceps thickness was not independently and significantly associated with BI score at discharge, BI score change, and BI efficiency.

## Discussion

We investigated the between intramuscular adipose tissue in the quadriceps at admission and recovery of ADL in older inpatients. Correlation analyses indicated that the intramuscular adipose tissue in the quadriceps at admission was negatively associated with recovery of ADL. Multiple regression analysis adjusted for confounding factors showed that intramuscular adipose tissue in the quadriceps was independently and negatively associated with recovery of ADL but that muscle mass was not associated with recovery of ADL. Taken together, our results indicate that greater intramuscular adipose tissue in the quadriceps at admission is more strongly associated with worse recovery of ADL than less muscle mass in older inpatients.

Similar relationships between intramuscular adipose tissue in the quadriceps and recovery of ADL were observed in the all participants, male, and female regression models, excluding the relationship between intramuscular adipose tissue in the quadriceps and ADL at discharge in the male model. Although there was no significant relationship between intramuscular adipose tissue in the quadriceps and ADL at discharge in the male model, standardized partial regression coefficients of these relationships in the all participants model (*β* = −0.13), female model (*β* = −0.15), and male model (*β* = −0.10) were approximately the same. A previous study^16^ reported no sex difference in perilipin2 expression in older patients. Considering this finding and our results, no sex differences may exist in the relationship between intramuscular adipose tissue in the quadriceps and recovery of ADL in older inpatients.

A more recent cross‐sectional study^19^ reported that greater intramuscular adipose tissue in the quadriceps is more strongly related to declines in ADL than less muscle mass in older inpatients. In this prospective study, a similar relationship was observed. In addition, previous studies reported that greater intramuscular adipose tissue in the quadriceps is more strongly associated with decreased muscle strength^5^, ^6^, ^7^ and decreased sit‐up–sit‐down,^5^, ^8^ stair ascent–stair descent,^9^ and gait abilities^6^, ^10^, ^11^ than less muscle mass. Our results also indirectly support these findings. Measurement of muscle mass does not always reflect the actual muscle mass because the area where muscle mass is measured includes both the muscle and intramuscular adipose tissue,^1^, ^14^ which potentially leads to overestimation of the muscle mass. These factors might have influenced our results (i.e. greater intramuscular adipose tissue is more strongly related to worse recovery of ADL than less muscle mass).

The Position Statements of the Sarcopenia Definition and Outcomes Consortium^34^ indicated that lean mass measured using dual‐energy X‐ray absorptiometry is not a good predictor of self‐reported mobility limitations, falls, hip fractures, and mortality. Based on this statement and the results of this study, greater intramuscular adipose tissue in the quadriceps, but not less muscle mass, in older inpatients is considered to be a predictor of worse recovery of ADL. This implies the importance of assessing the intramuscular adipose tissue in the quadriceps in older inpatients in a clinical setting.

Considering our results, lower amounts of intramuscular adipose tissue may be important for improving ADL in older inpatients. Englund *et al*.^35^ reported that physical activity and nutritional supplementation (whey protein and vitamin D) improved the amount of intramuscular adipose tissue in the thigh in community‐dwelling, mobility‐limited older people. In addition, Kitajima *et al*.^36^ confirmed the presence of reduced amounts of intramuscular adipose tissue in the lumbar muscles of patients with liver cirrhosis whose serum albumin concentration was improved following supplementation with branched‐chain amino acids. Overall, we speculate that an intervention aimed at improving physical activity and nutritional status is required to improve the amounts of intramuscular adipose tissue in the quadriceps of older inpatients. A further randomized controlled trial will be needed to reveal the causal relationship between intramuscular adipose tissue and recovery of ADL.

Our study had two strengths. First, this is the first study to show a negative relationship between the amount of intramuscular adipose tissue and recovery of ADL in older inpatients. These results indicate the importance of assessing intramuscular adipose tissue in older inpatients and the necessity of attention to intramuscular adipose tissue rather than muscle mass in order to improve ADL in older inpatients. Second, among the muscles of the upper and lower extremities, loss of muscle mass with ageing and disuse particularly occurs in the quadriceps,^37^, ^38^ and intramuscular adipose tissue in the quadriceps is closely related to motor function.^5^, ^6^, ^7^, ^8^, ^9^, ^10^, ^11^, ^19^ Therefore, targeting the quadriceps to examine the relationship between intramuscular adipose tissue and recovery of ADL is considered reasonable.

This study has three limitations. First, convenience sampling was performed in this study. Therefore, we were not able to completely exclude the influence of sampling bias on our results. Second, although magnetic resonance imaging and computed tomography provide more accurate measurements of intramuscular adipose tissue, we used ultrasound to assess this parameter because it is easily accessible, requires less time to perform, and is inexpensive.^39^ In addition, the validity of echo intensity measurements of intramuscular adipose tissue was demonstrated in studies using muscle biopsy^26^ and magnetic resonance imaging^27^ as gold standards. The last limitation is the generalizability of our results. A BI score <60 is considered a severely dependent condition of daily living.^40^ Considering the median (IQR) of the BI score at admission of the participants was 40.0 (20.0–60.0), almost all participants in the current study were in this condition. In addition, the median (IQR) of the GNRI score of the participants was 87.6 (80.0–93.8). Considering that a GNRI score of 82–92 indicates moderate malnutrition risk,^29^ the nutritional status of almost all participants in this study was poor. Taken together, our results were obtained from participants who had severely dependent daily living and poor nutritional status. Different findings may be obtained when groups with other characteristics are targeted.

## Conclusions

Our study indicates that greater intramuscular adipose tissue in the quadriceps at admission is more strongly related to worse recovery of ADL than less muscle mass in older inpatients. Greater intramuscular adipose tissue in the quadriceps in older inpatients is considered to be a predictor for the worse recovery of ADL, and intervening for greater intramuscular adipose tissue may be important for improving ADL in older inpatients.

## Author contributions

All the authors contributed in the conception and design of the study, or acquisition of data, or analysis and interpretation of data; in drafting the article; and in the final approval of the version to be submitted.

## Funding

This work was supported by the Japan Society for the Promotion of Science (JSPS) KAKENHI Grant Numbers JP17K18294 and JP20K19661.

## Conflict of interest

None declared.

## Supporting information


**Table S1.** Relationships between Barthel Index score at discharge and other variables in the male model (*n* = 183, R^2^ = 0.742, f^2^ = 2.876, statistical power = 1.000)

**Table S2.** Relationships between Barthel Index score change and other variables in the male model (*n* = 183, R^2^ = 0.205, f^2^ = 0.258, statistical power = 0.999)

**Table S3.** Relationships between Barthel Index efficiency and other variables in the male model (*n* = 183, R^2^ = 0.129, f^2^ = 0.148, statistical power = 0.947).

**Table S4.** Relationships between Barthel Index score at discharge and other variables in the female model (*n* = 221, R^2^ = 0.626, f^2^ = 1.674, statistical power = 1.000).

**Table S5.** Relationships between Barthel Index score change and other variables in the female model (*n* = 221, R^2^ = 0.245, f^2^ = 0.325, statistical power = 0.999).

**Table S6.** Relationships between Barthel Index efficiency and other variables in the female model (n = 221, R^2^ = 0.157, f^2^ = 0.186, statistical power = 0.987).
