# Supplementary material for: Intramuscular adipose tissue in the quadriceps is more strongly related to recovery of activities of daily living than muscle mass in older inpatients
Source: J Cachexia Sarcopenia Muscle. 2021 May 16;12(4):891–9. doi: 10.1002/jcsm.12713 (PMC8350216; doi:10.1002/jcsm.12713)
Supplement: Supplementary file 1 — Table S1. Relationships between Barthel Index score at discharge and other variables in the male model (n = 183, R2 = 0.742, f2 = 2.876, statistical power = 1.000) [file JCSM-12-891-s006.docx]

**Supporting Information Table S1. Relationships between Barthel Index score at discharge and other variables in the male model (n = 183, R^2^ = 0.742, f^2^ = 2.876, statistical power = 1.000)**

| **Variables** | **B** | **SE** | **95% Confidence interval of B** | **β** | **VIF** | **p-value** |
| --- | --- | --- | --- | --- | --- | --- |
| **Quadriceps echo intensity** | **−0.15** | **0.09** | **−0.32, 0.03** | **−0.10** | **2.54** | **0.10** |
| **Quadriceps thickness** | **−1.01** | **3.84** | **−8.58, 6.57** | **−0.02** | **3.00** | **0.79** |
| **Subcutaneous fat thickness of the thigh** | **0.14** | **7.90** | **−15.46, 15.73** | **0.00** | **1.39** | **0.99** |
| **Barthel Index score at admission** | **0.79** | **0.06** | **0.67, 0.90** | **0.67** | **1.62** | **<0.01** |
| **Age** | **0.09** | **0.18** | **−0.25, 0.44** | **0.02** | **1.32** | **0.60** |
| **Number of medications** | **−0.33** | **0.31** | **−0.94, 0.29** | **−0.05** | **1.19** | **0.30** |
| **C-reactive protein** | **−0.26** | **0.41** | **−1.07, 0.56** | **−0.03** | **1.36** | **0.54** |
| **Updated Charlson comorbidity index score** | **−1.01** | **0.51** | **−2.02, −0.01** | **−0.08** | **1.10** | **0.04** |
| **Food Intake Level Scale** | **2.25** | **0.73** | **0.80, 3.70** | **0.16** | **1.71** | **<0.01** |
| **Geriatric Nutritional Risk Index score** | **0.33** | **0.15** | **0.03, 0.62** | **0.12** | **2.01** | **0.03** |
| **Days from onset disease** | **−0.02** | **0.05** | **−0.11, 0.08** | **−0.03** | **7.68** | **0.75** |
| **Length of hospital stay** | **0.07** | **0.06** | **−0.05, 0.19** | **0.13** | **7.59** | **0.24** |
| **Number of rehabilitation therapy** | **1.91** | **0.79** | **0.36, 3.47** | **0.11** | **1.29** | **0.02** |
| **B, partial regression coefficient; SE, standard error; β, standardized partial regression coefficient; VIF, variance inflation factor** | | | | | | |
