# Supplementary material for: Intramuscular adipose tissue in the quadriceps is more strongly related to recovery of activities of daily living than muscle mass in older inpatients
Source: J Cachexia Sarcopenia Muscle. 2021 May 16;12(4):891–9. doi: 10.1002/jcsm.12713 (PMC8350216; doi:10.1002/jcsm.12713)
Supplement: Supplementary file 2 — Table S2. Relationships between Barthel Index score change and other variables in the male model (n = 183, R2 = 0.205, f2 = 0.258, statistical power = 0.999) [file JCSM-12-891-s004.docx]

**Supporting Information Table S2. Relationships between Barthel Index score change and other variables in the male model (n = 183, R^2^ = 0.205, f^2^ = 0.258, statistical power = 0.999)**

| **Variables** | **B** | **SE** | **95% Confidence interval of B** | **β** | **VIF** | **p-value** |
| --- | --- | --- | --- | --- | --- | --- |
| **Quadriceps echo intensity** | **−0.20** | **0.10** | **−0.40, −0.00** | **−0.22** | **2.54** | **0.04** |
| **Quadriceps thickness** | **−0.48** | **4.32** | **−9.01, 8.04** | **−0.01** | **3.00** | **0.91** |
| **Subcutaneous fat thickness of the thigh** | **−0.55** | **8.90** | **−18.11, 17.01** | **−0.01** | **1.39** | **0.95** |
| **Barthel Index score at admission** | **−0.24** | **0.07** | **−0.37, −0.11** | **−0.31** | **1.62** | **<0.01** |
| **Age** | **0.11** | **0.20** | **−0.28, 0.50** | **0.04** | **1.32** | **0.58** |
| **Number of medications** | **−0.26** | **0.35** | **−0.95, 0.43** | **−0.06** | **1.19** | **0.46** |
| **C-reactive protein** | **−0.26** | **0.47** | **−1.18, 0.66** | **−0.05** | **1.36** | **0.57** |
| **Updated Charlson comorbidity index score** | **−0.91** | **0.57** | **−2.04, 0.22** | **−0.11** | **1.10** | **0.11** |
| **Food Intake Level Scale** | **1.92** | **0.83** | **0.29, 3.55** | **0.21** | **1.71** | **0.02** |
| **Geriatric Nutritional Risk Index score** | **0.31** | **0.17** | **−0.02, 0.64** | **0.18** | **2.01** | **0.06** |
| **Days from onset disease** | **−0.01** | **0.05** | **−0.12, 0.09** | **−0.05** | **7.68** | **0.78** |
| **Length of hospital stay** | **0.05** | **0.07** | **−0.09, 0.18** | **0.13** | **7.59** | **0.50** |
| **Number of rehabilitation therapy** | **2.35** | **0.89** | **0.60, 4.11** | **0.21** | **1.29** | **0.01** |
| **B, partial regression coefficient; SE, standard error; β, standardized partial regression coefficient; VIF, variance inflation factor** | | | | | | |
