# Supplementary material for: Intramuscular adipose tissue in the quadriceps is more strongly related to recovery of activities of daily living than muscle mass in older inpatients
Source: J Cachexia Sarcopenia Muscle. 2021 May 16;12(4):891–9. doi: 10.1002/jcsm.12713 (PMC8350216; doi:10.1002/jcsm.12713)
Supplement: Supplementary file 3 — Table S3. Relationships between Barthel Index efficiency and other variables in the male model (n = 183, R2 = 0.129, f2 = 0.148, statistical power = 0.947). [file JCSM-12-891-s005.docx]

**Supporting Information Table S3. Relationships between Barthel Index efficiency and other variables in the male model (n = 183, R^2^ = 0.129, f^2^ = 0.148, statistical power = 0.947)**

| **Variables** | **B** | **SE** | **95% Confidence interval of B** | **β** | **VIF** | **p-value** |
| --- | --- | --- | --- | --- | --- | --- |
| **Quadriceps echo intensity** | **−0.00** | **0.00** | **−0.01, 0.00** | **−0.24** | **2.54** | **0.04** |
| **Quadriceps thickness** | **−0.03** | **0.08** | **−0.19, 0.13** | **−0.05** | **2.98** | **0.70** |
| **Subcutaneous fat thickness of the thigh** | **0.08** | **0.17** | **−0.25, 0.42** | **0.04** | **1.38** | **0.63** |
| **Barthel Index score at admission** | **−0.00** | **0.00** | **−0.01, 0.00** | **−0.15** | **1.59** | **0.09** |
| **Age** | **0.00** | **0.00** | **−0.01, 0.01** | **0.03** | **1.27** | **0.74** |
| **Number of medications** | **−0.00** | **0.01** | **−0.02, 0.01** | **−0.02** | **1.19** | **0.83** |
| **C-reactive protein** | **−0.01** | **0.01** | **−0.03, 0.01** | **−0.10** | **1.36** | **0.24** |
| **Updated Charlson comorbidity index score** | **−0.02** | **0.01** | **−0.04, 0.00** | **−0.14** | **1.09** | **0.06** |
| **Food Intake Level Scale** | **0.02** | **0.02** | **−0.01, 0.05** | **0.11** | **1.68** | **0.25** |
| **Geriatric Nutritional Risk Index score** | **0.00** | **0.00** | **−0.01, 0.01** | **0.04** | **2.01** | **0.69** |
| **Days from onset disease** | **−0.00** | **0.00** | **−0.00, 0.00** | **−0.15** | **1.27** | **0.07** |
| **Number of rehabilitation therapy** | **0.01** | **0.02** | **−0.02, 0.05** | **0.06** | **1.28** | **0.50** |
| **B, partial regression coefficient; SE, standard error; β, standardized partial regression coefficient; VIF, variance inflation factor** | | | | | | |
