# Supplementary material for: Intramuscular adipose tissue in the quadriceps is more strongly related to recovery of activities of daily living than muscle mass in older inpatients
Source: J Cachexia Sarcopenia Muscle. 2021 May 16;12(4):891–9. doi: 10.1002/jcsm.12713 (PMC8350216; doi:10.1002/jcsm.12713)
Supplement: Supplementary file 4 — Table S4. Relationships between Barthel Index score at discharge and other variables in the female model (n = 221, R2 = 0.626, f2 = 1.674, statistical power = 1.000). [file JCSM-12-891-s002.docx]

**Supporting Information Table S4. Relationships between Barthel Index score at discharge and other variables in the female model (n = 221, R^2^ = 0.626, f^2^ = 1.674, statistical power = 1.000)**

| **Variables** | **B** | **SE** | **95% Confidence interval of B** | **β** | **VIF** | **p-value** |
| --- | --- | --- | --- | --- | --- | --- |
| **Quadriceps echo intensity** | **−0.20** | **0.08** | **−0.36, −0.04** | **−0.15** | **2.17** | **0.02** |
| **Quadriceps thickness** | **−1.67** | **4.57** | **−10.69, 7.35** | **−0.02** | **2.45** | **0.72** |
| **Subcutaneous fat thickness of the thigh** | **−3.45** | **5.88** | **−15.04, 8.15** | **−0.03** | **1.65** | **0.56** |
| **Barthel Index score at admission** | **0.67** | **0.07** | **0.53, 0.81** | **0.54** | **1.88** | **<0.01** |
| **Age** | **−0.05** | **0.20** | **−0.44, 0.35** | **−0.01** | **1.30** | **0.82** |
| **Number of medications** | **−0.36** | **0.35** | **−1.05, 0.34** | **−0.05** | **1.10** | **0.32** |
| **C-reactive protein** | **−0.51** | **0.55** | **−1.59, 0.56** | **−0.04** | **1.17** | **0.35** |
| **Updated Charlson comorbidity index score** | **−2.82** | **0.65** | **−4.10, −1.54** | **−0.20** | **1.13** | **<0.01** |
| **Food Intake Level Scale** | **2.81** | **0.94** | **0.96, 4.66** | **0.16** | **1.51** | **<0.01** |
| **Geriatric Nutritional Risk Index score** | **0.17** | **0.17** | **−0.16, 0.50** | **0.06** | **1.79** | **0.31** |
| **Days from onset disease** | **−0.05** | **0.04** | **−0.14, 0.03** | **−0.10** | **3.36** | **0.21** |
| **Length of hospital stay** | **0.12** | **0.04** | **0.03, 0.20** | **0.22** | **3.34** | **0.01** |
| **Number of rehabilitation therapy** | **0.40** | **0.84** | **−1.25, 2.05** | **0.02** | **1.18** | **0.64** |
| **B, partial regression coefficient; SE, standard error; β, standardized partial regression coefficient; VIF, variance inflation factor** | | | | | | |
