# Supplementary material for: Intramuscular adipose tissue in the quadriceps is more strongly related to recovery of activities of daily living than muscle mass in older inpatients
Source: J Cachexia Sarcopenia Muscle. 2021 May 16;12(4):891–9. doi: 10.1002/jcsm.12713 (PMC8350216; doi:10.1002/jcsm.12713)
Supplement: Supplementary file 5 — Table S5. Relationships between Barthel Index score change and other variables in the female model (n = 221, R2 = 0.245, f2 = 0.325, statistical power = 0.999). [file JCSM-12-891-s001.docx]

**Supporting Information Table S5. Relationships between Barthel Index score change and other variables in the female model (n = 221, R^2^ = 0.245, f^2^ = 0.325, statistical power = 0.999)**

| **Variables** | **B** | **SE** | **95% Confidence interval of B** | **β** | **VIF** | **p-value** |
| --- | --- | --- | --- | --- | --- | --- |
| **Quadriceps echo intensity** | **−0.22** | **0.09** | **−0.39, −0.06** | **−0.23** | **2.17** | **0.01** |
| **Quadriceps thickness** | **−2.12** | **4.73** | **−11.44, 7.20** | **−0.04** | **2.45** | **0.66** |
| **Subcutaneous fat thickness of the thigh** | **−3.03** | **6.08** | **−15.01, 8.96** | **−0.04** | **1.65** | **0.62** |
| **Barthel Index score at admission** | **−0.32** | **0.07** | **−0.46, −0.17** | **−0.35** | **1.88** | **<0.01** |
| **Age** | **0.05** | **0.21** | **−0.36, 0.45** | **0.02** | **1.30** | **0.82** |
| **Number of medications** | **−0.56** | **0.37** | **−1.28, 0.16** | **−0.10** | **1.10** | **0.13** |
| **C-reactive protein** | **−0.32** | **0.56** | **−1.43, 0.80** | **−0.04** | **1.17** | **0.58** |
| **Updated Charlson comorbidity index score** | **−2.68** | **0.67** | **−4.01, −1.36** | **−0.26** | **1.13** | **<0.01** |
| **Food Intake Level Scale** | **3.11** | **0.97** | **1.19, 5.02** | **0.24** | **1.51** | **<0.01** |
| **Geriatric Nutritional Risk Index score** | **0.17** | **0.17** | **−0.17, 0.51** | **0.08** | **1.79** | **0.32** |
| **Days from onset disease** | **−0.05** | **0.04** | **−0.13, 0.04** | **−0.12** | **3.36** | **0.28** |
| **Length of hospital stay** | **0.11** | **0.04** | **0.03, 0.20** | **0.30** | **3.34** | **0.01** |
| **Number of rehabilitation therapy** | **0.72** | **0.87** | **−0.99, 2.43** | **0.06** | **1.18** | **0.41** |
| **B, partial regression coefficient; SE, standard error; β, standardized partial regression coefficient; VIF, variance inflation factor** | | | | | | |
