# Supplementary material for: Intramuscular adipose tissue in the quadriceps is more strongly related to recovery of activities of daily living than muscle mass in older inpatients
Source: J Cachexia Sarcopenia Muscle. 2021 May 16;12(4):891–9. doi: 10.1002/jcsm.12713 (PMC8350216; doi:10.1002/jcsm.12713)
Supplement: Supplementary file 6 — Table S6. Relationships between Barthel Index efficiency and other variables in the female model (n = 221, R2 = 0.157, f2 = 0.186, statistical power = 0.987). [file JCSM-12-891-s003.docx]

**Supporting Information Table S6. Relationships between Barthel Index efficiency and other variables in the female model (n = 221, R^2^ = 0.157, f^2^ = 0.186, statistical power = 0.987)**

| **Variables** | **B** | **SE** | **95% Confidence interval of B** | **β** | **VIF** | **p-value** |
| --- | --- | --- | --- | --- | --- | --- |
| **Quadriceps echo intensity** | **−0.00** | **0.00** | **−0.01, 0.00** | **−0.21** | **2.14** | **0.02** |
| **Quadriceps thickness** | **0.07** | **0.09** | **−0.11, 0.24** | **0.07** | **2.44** | **0.47** |
| **Subcutaneous fat thickness of the thigh** | **−0.11** | **0.12** | **−0.33, 0.12** | **−0.08** | **1.63** | **0.36** |
| **Barthel Index score at admission** | **−0.00** | **0.00** | **−0.01, 0.00** | **−0.18** | **1.80** | **0.04** |
| **Age** | **0.00** | **0.00** | **−0.01, 0.01** | **−0.01** | **1.29** | **0.91** |
| **Number of medications** | **−0.02** | **0.01** | **−0.03, −0.01** | **−0.18** | **1.10** | **0.01** |
| **C-reactive protein** | **0.00** | **0.01** | **−0.02, 0.02** | **0.00** | **1.17** | **0.99** |
| **Updated Charlson comorbidity index score** | **−0.03** | **0.01** | **−0.06, −0.01** | **−0.18** | **1.13** | **0.01** |
| **Food Intake Level Scale** | **0.04** | **0.02** | **−0.00, 0.07** | **0.15** | **1.50** | **0.06** |
| **Geriatric Nutritional Risk Index score** | **0.00** | **0.00** | **−0.01, 0.01** | **0.02** | **1.79** | **0.85** |
| **Days from onset disease** | **−0.00** | **0.00** | **−0.00, 0.00** | **−0.11** | **1.08** | **0.09** |
| **Number of rehabilitation therapy** | **0.02** | **0.02** | **−0.01, 0.05** | **0.08** | **1.15** | **0.28** |
| **B, partial regression coefficient; SE, standard error; β, standardized partial regression coefficient; VIF, variance inflation factor** | | | | | | |
